# Influence of Anticaking Agents and Storage Conditions on Quality Characteristics of Spray Dried Apricot Powder: Shelf Life Prediction Studies Using Guggenheim-Anderson-de Boer (GAB) Model

**DOI:** 10.3390/foods12010171

**Published:** 2022-12-29

**Authors:** Omar Bashir, Syed Zameer Hussain, Kashif Ameer, Tawheed Amin, Beenish, Isam A. Mohamed Ahmed, Moneera O. Aljobair, Gousia Gani, Shakeel Ahmad Mir, Qudsiya Ayaz, Nowsheen Nazir

**Affiliations:** 1 Division of Food Science and Technology, Sher-e-Kashmir University of Agricultural Sciences and Technology of Kashmir, Srinagar 190025, India; 2 Department of Food Technology and Nutrition, Lovely Professional University, Phagwara 144402, India; 3 Institute of Food Science and Nutrition, University of Sargodha, Sargodha 40100, Pakistan; 4 Department of Food Science and Technology, Faculty of Agriculture, University of Khartoum, Shambat 13314, Sudan; 5 Department of Food Science and Nutrition, College of Food and Agricultural Sciences, King Saud University, P.O. Box 145111, Riyadh 11451, Saudi Arabia; 6 Department of Physical Sport Science, College of Education, Princess Nourah bint Abdulrahman University, P.O. Box 84428, Riyadh 11671, Saudi Arabia; 7 Division of Agricultural Statistics, Sher-e-Kashmir University of Agricultural Sciences and Technology of Kashmir, Srinagar 190025, India; 8 Division of Fruit Science, Sher-e-Kashmir University of Agricultural Sciences and Technology of Kashmir, Srinagar 190025, India

**Keywords:** accelerated storage, flowability, glass transition temperature, hygroscopicity

## Abstract

Apricot powder was developed through spray drying using gum arabic as an encapsulating material at a concentration of 19%. Inlet air temperature, feed total soluble solids (TSS), feed flow rate, and atomization speed were 190 °C, 23.0 °C, 300.05 mL/h, and 17,433 rpm, respectively. This study was therefore conducted to investigate the influence of anticaking agents (tricalcium phosphate and silicon dioxide) and storage conditions (ambient and accelerated) on physicochemical, micrometric, and thermal characteristics of spray-dried apricot powder (SDAP) packaged in aluminum laminates. Both tricalcium phosphate (TCP) and silicon dioxide (SiO_2_) improved the shelf life and quality of SDAP, with TCP being more effective, since a lower increase in water activity (a_w_), moisture content, degree of caking, hygroscopicity, and rehydration time was observed in TCP-treated samples followed by SiO_2_-treated samples than the control. Furthermore, flowability, glass transition temperature (Tg), and sticky-point temperature (Ts) of SDAP tended to decrease in a significant manner (*p* < 0.05) under both storage conditions. However, the rate of decrease was higher during accelerated storage. The water activity of treated samples under ambient conditions did not exceed 0.60 and had a total plate count within the permissible range of 40,000 CFU/g, indicating shelf stability of the powder. The predicted shelf life of powder obtained from the Guggenheim–Anderson–de Boer (GAB) model and experimental values were very similar, with TCP-treated samples having a predicted shelf life of 157 days and 77 days under ambient and accelerated storage conditions, respectively. However, the respective experimental shelf life under the same conditions was 150 and 75 days, respectively. Similarly, the predicted shelf life of SiO_2_-treated samples under ambient and accelerated storage was 137 and 39 days, respectively, whereas the experimental values were 148 and 47 days, respectively. In conclusion, TCP proved more effective than SiO_2_ at preserving shelf life by preventing moisture ingress.

## 1. Introduction

Apricots (*Prunus armeniaca*) are appreciated by consumers for their flavor, juiciness, and sweetness. It is classified as perishable naturally, with the fruit having a very short shelf-life of 3–5 days at ambient temperature, and thus the acceptability regarding fresh consumption tends to decrease, minimizing the chances of usage of the perishable fruit for further processing [1]. Hence, it is also necessary to transform the fruit to value-added commercial products prior to loss of fruit freshness and usefulness. Fruits are usually subjected to various preservation and processing techniques that may ensure shelf-life enhancement of perishable fruits and vegetables like apricot. Powder production through dehydration is a good choice included in fruit-preservation methods since it leads to reduction of moisture, microbial growth inhibition, and enzymatic inactivation [2]. One such dehydration technique is the use of spray-drying technology [3,4]. However, there are many challenges in the handling, production, and storage of fruit powders. These include stickiness, thermoplasticity, and hygroscopicity, particularly at higher temperature and humidity levels [5]. As a result, powder may also undergo processes of bridging, agglomeration, compaction, and liquefaction, which may consequently lead to caking issues in powdered products [6]. Thus, it may reduce the shelf stability by hampering the quality of the product as well as functional properties, such as poor rehydration of powdered products, low degree of solubility, and dispersibility in conjunction with low microbial quality [7].

Stickiness of fruit powders is mainly due to low-molecular-weight sugars and some organic acids with low glass transition temperature (Tg) and high hygroscopicity values. Fruit powders with high sugar content tend to be more hygroscopic in an amorphous state, thereby leading to caking and reduction in flow characteristics [8]. Studies on food-product quality changes with respect to time are indispensable for ensuring the compliance of food products in terms of international food standards. Various intrinsic and extrinsic factors also influence the cohesive and adhesive forces that consequently govern the complex stickiness phenomenon in foods. Intrinsic factors include viscoelastic properties and surface tension. Extrinsic factors include the humidity and temperature. Various factors including environmental conditions to which the powder products are exposed and properties of the packaging material, such as permeability to oxygen, moisture, and light, affect the storage stability and thus characteristics of fruit powder [9].

Thus, it is also necessary to carry out some useful unit operations regarding dehydrated fruit powders to protect them against moisture ingress, oxygen penetration, and volatile flavoring compounds and color loss. The use of packaging materials for powdered food products with better barrier properties against these extreme conditions is a useful approach in this context. Amongst the various packaging materials, aluminum-laminated packages (ALP) have better heat sealability and provide an excellent barrier to light and oxygen permeability [10]. Furthermore, anticaking agents are usually added to powdered products during production for improving storage stability [11]. Tricalcium phosphate (TCP) is one of the most commonly employed anticaking agents used in the formulation of food products. TCP not only has a functional role but is also employed as a nutrient supplement for the provision of additional phosphorus and dietary calcium to consumers. TCP involves applications not merely for producing dry and powdered products but may also be utilized for the formulation of liquid/moist items, such as infant formula and yogurt. Silicon dioxide (SiO_2_) is known by another common name: silica. It exists in nature and is part of the chemical composition of several plants, such as alfalfa, brown rice, beets, and green leafy vegetables. Anticaking agents such as SiO_2_ and TCP increase the stability of powders through the alteration of lattice patterns existing in the structural configuration of molecular structure, thereby causing the glassy–rubbery transition phase to be delayed by competing with the host powder and acting as a protective barrier against moisture [7]. Therefore, this study was carried out to evaluate the influence of anticaking agents and storage conditions on physicochemical, micrometric, and thermal characteristics of spray-dried apricot-pulp powder. Furthermore, accelerated storage involving higher humidity and temperature values may be used to develop the relationships between moisture ingress and storage time to predict the shelf stability of a dried food product [11]. Therefore, the prediction model (GAB model) was developed for predicting the shelf-life stability of SDAP packaged in ALP under both ambient and accelerated storage conditions for a period of 180 days.

## 2. Materials and Methods

### 2.1. Materials

The *Halman* variety of fresh apricot was employed for recovering pulp and apricot-variety procurement was carried out in Krishi Vigyan Kendra, SKUAST-K, Kargil, Ladakh, India. Pectinase enzyme (Pectinex Ultra SP-L) was used for the juice-extraction process. During spray drying, gum arabic (GA 20) sourced from Himedia (India) was employed as the carrier agent. For storage studies, aluminum-laminated polyethylene (ALP) (0.5 kg capacity with a thickness of 0.05 mm) was used as packaging material and was purchased from an Indian supplier of packaging material based in India (Omflex: Flexible Packaging Material, New Delhi, India). Purchasing of food-grade anticaking agents viz. TCP and SiO_2_ was carried out by the Spectrum Chemical Mfg Corp. (Gardena, CA, USA).

### 2.2. Spray Drying to Prepare Apricot Powder

SDAP was prepared using a pilot plant spray dryer (SMST, Calcutta, India) with a co-current air flow. In order to carry out the spray-drying procedure, the fruit pulp was first treated with pectinase enzyme for juice extraction with 0.90% (*w*/*w*) pectinase enzyme at an operational temperature of 44 °C for a time period of 300 min. Some preliminary studies were performed in order to select the pectinase enzyme concentration [12]. The clarified juice was spray dried after optimizing the process conditions using a statistical model of response surface methodology at a gum arabic concentration, inlet air temperature, feed total soluble solids (TSS), feed flow rate, and atomization speed of 19%, 190 °C, 23.0 °C, 300.05 mL/h, and 17,433 rpm, respectively.

### 2.3. SDAP Storage

Briefly, 20 g SDAP was added to SiO_2_ and TCP separately, each at a concentration of 0.017 kg/kg (based on some preliminary trials). Packing was then carried out using aluminum-laminated polyethylene (ALP) pouches with 0.5 kg capacity and a thickness of 0.05 mm followed by heat sealing. Storage of the powder was then carried out under two different storage conditions, ambient storage (25 ± 2 °C, 50 ± 2% relative humidity) and accelerated storage conditions (40 ± 2 °C, 90 ± 2% RH), for 180 days. The temperature and relative humidity conditions during storage were maintained by placing the pouches containing the samples in an incubator. SDAP samples without the addition of anticaking agents were taken as control.

#### 2.3.1. Physicochemical Properties

SDAP was analyzed every month for the first four months of storage and then every 20 days for the next two months for the following physicochemical parameters over a period of 180 days of storage.

##### Initial and Critical Moisture Content and Water-Activity Determination

Moisture content of the samples was determined by following standard AOAC procedures [13]. Initial moisture content was determined by means of a moisture analyzer (Kern, DBS-BA-def-1714, Lohmar, Germany) at a temperature of 105 °C using an amount of 2 g of sample. Water-activity (a_w_) estimation was performed using a smart water-activity meter (Water Activity Analyzer, Decagon Devices Inc., Pullman, WA, USA) [12]. The sample was filled (3/4) in the cup of the water-activity meter. The instrument was calibrated and then readings were recorded. All measurements were performed in triplicates. Critical water activity was obtained from the literature review and critical moisture content was calculated by means of a generated polynomial equation.

##### Degree of Caking

The reported method by Ramachandran and Rao [14] was used to determine the degree of caking. In brief, spray-dried powder was taken after taking a measured amount (5 g) followed by pouring into a sieve (Zebra, Malaysia) with a 25.40 μm opening. The rigorous shaking of powder was carried out until no further passing of powder through the sieve. The weight of the remaining powder on the sieve was determined and recorded. Equation (1) was used for calculation of the degree of caking;

(1) $$\mathrm{DC}\;=\frac{c}{d}\times 100\;$$

where d (g) denotes to the amount of powder used for sieving and c (g) is indicative of the amount of powder left on the sieve after sieving.

#### 2.3.2. Micrometric Properties

##### Flowability

Samples of powder were subjected to measurement of flowability according to the procedures described by Jinapong et al. [15]. The Carr’s index (CI) in terms of bulk and tapped densities to express flowability was calculated (Equation (2)). CI values of powder < 15 were regarded as very good, whereas the range of 15–20 indicated good powder and values of CI of 20–35, 35–45, and >45 were indicative of fair, bad, and very bad flowability of the powder products, respectively.

(2) $$\mathrm{Carr}’s\;\mathrm{index}\;\left(CI\right)=\frac{\rho_{t}-\rho_{b}}{\rho_{t}}\times 100\;$$

where

ρ_t_ = tapped density
ρ_b_ = bulk density

##### Hygroscopicity

Spray-dried powdered samples were analyzed to determine their hygroscopicity as per the reported method by Cai and Corke [16]. Briefly, 2 g sample were placed at 25 °C in an airtight plastic container with dimensions of 40 × 20 × 25 cm. The plastic containers filled with samples were also added to Na_2_SO_4_ saturated solution (81% RH). After completion of a time period of 1 week, the samples were subjected to measurement of hygroscopic moisture through weighing and the recorded measurement was expressed in terms of g of moisture/100 g dry solids (g/100 g).

##### Rehydration Time and Rehydration Ratio

The rehydration time of the powder sample was determined following the procedures of Goula and Adamopoulos [17], whereas the reported method of Puttongsiri et al. [18] was utilized for determining rehydration ratio as per Equation (3), given below.

(3) $$\mathrm{Rehydration}\;\mathrm{ratio}=\frac{\mathrm{Weight}\;\mathrm{of}\;\mathrm{rehydrated}\;\mathrm{sample}\;\left(g\right)}{\mathrm{Weight}\;\mathrm{of}\;\mathrm{dried}\;\mathrm{sample}\;\left(g\right)}$$

#### 2.3.3. Color-Value Determination

The color values of the control and SiO_2_- and TCP-incorporated SDAP samples were determined by Hunter Lab Colorimeter (Model No. SN3001476, Accuracy Micro-Sensors, New York, NY, USA) according to the reported method of Rai et al. [19].

#### 2.3.4. Microbiological Analysis

The reported method of Liu et al. [20] was employed for determination of the total plate count. Briefly, 1 g of powder sample was homogenized at 5000 g using a homogenizer (Ultra-turrax T25 blade-type homogenizer; Ika-Werk, Staufen, Germany) in 9.0 mL sterile 0.1% peptone water for 30 s and then serial dilution was carried out. Aliquots in a quantity of 1 mL were plated, and nutrient agar (CM003; Oxoid Ltd., Basingstoke, Hampshire, UK) and Malt Extract Agar (CM 0099; Oxoid), being suitable media for the growth of total aerobic bacteria and fungi, respectively, were used for media preparation. Plated aliquots for total aerobic bacteria were incubated at 37 °C for 24 h, whereas fungi-plated aliquots were incubated at 25 °C for 5 days. A colony counter (LM-10; Analab, New Delhi, India) was used to enumerate the microbial population, and the counts of the microorganisms were recorded and the readings were taken from 10^−3^ serial dilution.

#### 2.3.5. Storage-Life Prediction of SDAP

Moisture content in the foods usually governs the a_w_ of foodstuffs. For the purpose of the characterization of moisture transfer through packaging material, usually moisture sorption isotherm relationships are employed that establish the relationship between water activity and moisture content of foods. Typical mathematical expressions employed for study/modeling of water activity of foodstuffs include the GAB (Guggenheim–Anderson–de Boer) and the Halsey, Oswin, I & C (Iglesias and Chirife) models. Usually, the application of moisture sorption isotherms of foods helps with the following: (1) determination of dehydration and concentration processes; (2) to aid in the formulation of complex food mixtures; (3) identification of sustainable packaging materials; (4) classification of microorganisms of potential interest on the basis of moisture content, which will restrict their growth; and (5) prediction of chemical and physical stability of foods as function of moisture content. The isotherm curve fitting is usually carried out through the GAB model, which involves a multilayer equation with two constants providing the mathematical description of foods in relation to a maximum value of up to 0.94. The curve linearity and adjoining points together help with the estimation of the water-vapor transmission rate, permeability, and shelf life of moisture-sensitive/humidity-sensitive powders together with package information. Shelf-life evaluation at regular intervals is usually conducted in longer periods and is regarded as expensive in a general manner. Moreover, shelf-life testing involves mandatory assessment through the product-development cycle and process and reformulation changes. Measurement of the rate of change of moisture content was carried out in terms of d_X_/dθ of powder with respect to storage time (θ) as per Equation (4).

(4) $$W_{s}\frac{\mathrm{dx}}{d\theta }=KA_{p}\left(\mathrm{Rhp}*-\mathrm{awp}*\right)$$

where dry weight of apricot powder inside packaging material is denoted by W_s_ (kg); water-saturation vapor pressure is shown by p* (Pa); the storage temperature is denoted by T (°C); the relative humidity of the storage environment is shown by Rh; packaging material permeability is shown by K (kg water/m^2^/day/Pa); A_p_ (m^2^) denotes the packaging-material surface area through which the permeation of water vapor occurs; aw denotes the water activity of powdered samples at T (°C); and powder moisture content is shown by X (kg H*_2_*O)/kg dry solids) after a certain number of day of storage time (θ).

Equation (4) was solved to elucidate the graphical relationship between the moisture content X (kg H_2_O)/kg dry solids) and storage time in terms of days (θ). The observed values were subjected to comparison with the experimental moisture-content values. Equation (4) was solved to determine the storage time (θ) required for powder moisture content to increase from an initial value (Xi: kg H_2_O)/kg dry solids) to its critical value (Xc: kg H_2_O)/kg dry solids).

#### 2.3.6. Determination of Glass Transition (Tg) and Sticky-Point (Ts) Temperatures

Powders were subjected to determination of glass transition (T_g_) and sticky-point (T_s_) temperatures by means of a differential scanning calorimeter (Q600 SDT and Q20 DSC; TAI). Briefly, 5 mg powder sample were placeed in an aluminum pan, followed by equilibration at 25 °C and 32% RH using MgCl_2_-saturated solution for 1 week. The calibration of the equipment was done using indium and the sample was first heated at a temperature ranging from 10 to 180 °C at a 10 °C/min rate followed by cooling to 25 °C at the rate of 10 °C/min. Calculation of glass transition midpoint values as well as sticky-point temperatures of the sample was carried out by employing software called Thermal Advantage (version 1.1A), taking an empty pan as the reference.

### 2.4. Statistical Analysis

In order to conduct data analysis, SPSS statistics (v. 16. Inc, Chicago, IL, USA) software was utilized. Data were analyzed using three-way ANOVA as a function of storage duration, storage conditions, and anticaking agents, and a general linear model was used for data analysis. Duncan’s multiple-range test was employed to determine the differences between the means at a significance level of *p* < 0.05. Triplicate measurements of all experiments were recorded.

## 3. Results and Discussion

### 3.1. Moisture Content

**Figure 1:**
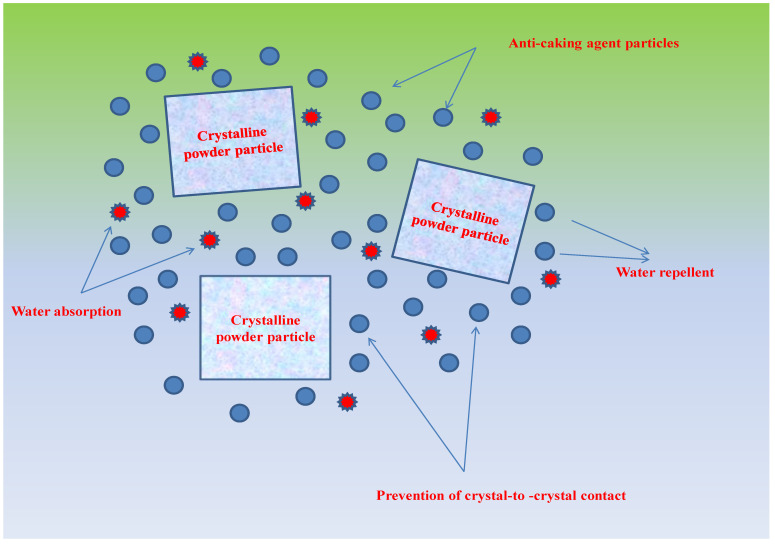
Mechanisms by which anticaking agents influence the water–solid interactions, preventing an increase in moisture gain.

One of the most important determinants of powder quality is moisture-content determination, as moisture content is indicative of the water composition in food systems [21]. The influence of anticaking agents (TCP and SiO_2_), storage conditions, and storage duration on the moisture content of SDAP is shown in Table 1. It was evident from the results that anticaking agents, storage period, and storage conditions exhibited a significant (*p* < 0.05) effect on moisture content. Generally, the moisture content of all the powder samples increased with the storage duration under both storage conditions, i.e., ambient and accelerated. However, a higher increase was observed under accelerated storage conditions, with moisture content increasing from 5.61 ± 0.03% to 11.99 ± 0.01% compared to ambient storage, respectively, whereas it increased from 5.61 ± 0.03% to 9.98 ± 0.03%, respectively, for control. The increasing tendency of the moisture content with the corresponding increase in storage period may be attributed to water-vapor migration through the packaging material in the storage environment [22]. However, anticaking agents TCP and SiO_2_ may have protected the powder from moisture uptake into the food product inside (Table 1). Under ambient and accelerated conditions, both TCP- and SiO_2_-treated samples exhibited significantly (*p* < 0.05) lower moisture content at the end of storage compared to control. The results revealed the protective effects of anticaking agents in terms of formation of a moisture-protective barrier existing between the particulate matter of the powdered samples [7] (Figure 1). Anticaking agents have the potential to rapidly absorb excess moisture up to 2.50 times their weight and still withhold a free flow, in addition to absorbing non-polar organic compounds and oils, by encapsulating powder particles [11]. Moreover, anticaking agents exhibit smaller particle sizes compared to those of host powders and usually have a role in covering the surface of crystals and reducing the contact points of crystals [7] (Figure 1). By creating a physical barrier, anticaking agents also likely have a role in the interruption of the liquid–film continuity formed between particles, with a corresponding rise in the moisture content and therefore causing a reduction in the strength of solid bridges under a period of storage intervals [23]. Lipasek et al. [23] reported the better protective role of TCP in preventing moisture ingress than SiO_2_, thereby supporting our results.

### 3.2. Water Activity

Table 1 shows the influence of storage conditions, storage duration, and anticaking agents (TCP and SiO_2_) on the water activity (a_w_) of SDAP. Control samples exhibited a significant (*p* < 0.05) increase in a_w_ during storage from an initial value of 0.28 ± 0.03 to 0.71 ± 0.01 and from 0.28 ± 0.03 to 0.88 ± 0.03 during ambient and accelerated conditions, respectively. Our results are consistent with the findings of Dak et al. [24] for pomegranate-aril powder. Between powder particles, the moisture-protective barrier is usually formed by anticaking agents by causing prevention of moisture adsorption, thus not allowing an increase in a_w_ [7]. Since the a_w_ of samples incorporated with TCP and SiO_2_ was ≤ 0.65 under ambient conditions, it can be inferred that powder samples exhibited increased stability against microbial contamination through microbial invasions, lipid oxidation, hydrolytic reactions, auto-oxidation browning, and enzymatic activity [25]. Furthermore, the a_w_ of all the samples under accelerated conditions incorporated with either TCP or SiO_2_ was also found to be significantly (*p* < 0.05) lower than the control. However, the a_w_ of samples under accelerated storage incorporated with both TCP and SiO_2_ was 0.72 ± 0.04 and 0.73 ± 0.04, respectively, signifying the powders to be relatively less stable after completion of the storage interval, since microbial and chemical activities may be induced at higher a_w_ values [25]. Our findings are consistent with those of the results of Gabas et al. [26], who reported an increasing tendency in water activity with corresponding increases in the temperature, relative humidity (RH), and storage time.

### 3.3. Degree of Caking

It was evident from the results of Table 1 that all parameters, such as storage conditions, storage duration, and anticaking agents, exhibited a significant (*p* < 0.05) effect on degree of caking. A significant increase in the degree of caking was observed in control under ambient conditions (10.19 ± 0.03% to 20.59 ± 0.03%) and accelerated storage conditions (10.19 ± 1.09 to 24.76 ± 1.08%). The higher increase in the degree of caking under accelerated storage conditions might have been due to the higher rates of moisture transmission at higher relative humidity values. The stored powders under such storage conditions had an increased tendency toward easy moisture absorption from their surroundings, resulting in powder stickiness and eventually leading to caking [14]. Furthermore, a rising trend in moisture content may cause a decrease in powder glass transition temperature (T_g_), which leads to plasticizing of sugar-rich powder particles, in turn leading to caking [8]. Reports published by Fabra et al. [8] and Goula et al. [17] also detail a stepwise increase in the gradual manner in the degree of caking with a corresponding declining trend in Tg for grapefruit and tomato powders, respectively, over storage time.

Anticaking agents have been reported to be involved in underpinning mechanisms of host-powder particle separation of those competing for moisture. Hence, this leads to inhibition of the caking phenomenon. The samples incorporated with TCP and SiO_2_ exhibited a lower degree of caking under both ambient (12.36 ± 1.07% and 13.03 ± 1.03%, respectively) and accelerated storage conditions (14.80 ± 1.03 and 15.24 ± 1.03%, respectively) compared to control over the storage period. This might be ascribed to the improvement in the stability of the powders owing to the increase in T_g_ [7]. It has been reported that the T_g_ increases with an increase in the molecular weight of polymers [27], and anticaking agents, being high molecular-weight components, may have resulted in the increases in T_g_. TCP, with a higher molecular weight than SiO_2_, imparted better anticaking activity, as is evident from the results in Table 1. Thus, TCP was found to be more effective than SiO_2_ in preventing caking in powder samples. Similar results have been reported by Phanindrakumar et al. [28] for pineapple powder.

### 3.4. Flowability

Carr’s index (CI) serves as an indicator to express degree of flowability. It is one of the most important quality attributes for gauging powder features playing an integral role of pertinent significance in various processes, such as the mobility of powdered products and transportation, dosage, and mixing [29]. The higher values of Carr’s index indicate the poor flowability. All storage parameters, including storage period, storage conditions, and anticaking agents, exhibited significant effects (*p* < 0.05) on flowability. All the powder samples at the commencement of the storage period had lower CI values of 22.35 ± 1.01%, which is indicative of fair powder flow (Table 2). The flowability of all the samples, either treated or control, showed a significant (*p* < 0.05) decreasing tendency with storage time. The samples incorporated with anticaking agents (TCP and SiO_2_) under ambient conditions (32.65 ± 1.03% and 33.70 ± 2.02%, respectively) had better flowability compared to control (34.26 ± 2.02%); however, all treated and control samples had fair flow. Under accelerated storage conditions, the decrease in flowability was observed in both the treated and control samples; however, the effect was more pronounced in control (36.98 ± 2.31%), signifying bad flow of the powder. The lower flowability of the powder under accelerated conditions was attributed to the higher rates of moisture transmission, which may have led to lowering the T_g_, thus making the powder lumpy and sticky [8,14]. As indicated in Table 1, the moisture content of samples stored under accelerated storage conditions was higher compared to ambient conditions, which was responsible for the decrease in T_g_ of the powder, thus reducing flowability. T_g_ has been found to have an inverse relationship with the moisture content, thus supporting our results [27].

### 3.5. Hygroscopicity

It is evident from the results of Table 2 that all parameters, such as storage conditions, storage duration, and anticaking agents, exhibited a significant (*p* < 0.05) effect on hygroscopicity. Generally, the hygroscopicity of all spray-dried samples increased with storage duration under both storage conditions, with a higher increase under accelerated storage conditions than ambient. The increase in hygroscopicity of powder samples that were subjected to packaging using ALP pouches under accelerated conditions might have been due to the water-vapor migration from the storage environment into the packaging material [22]. Powder samples with high hygroscopicity have the capability to absorb more moisture from the surrounding environment easily, resulting in powder caking [14]. An increase in moisture content decreased the T_g_ of the samples, which may have resulted in the production of powders with lower stickiness values [7]. The samples incorporated with TCP and SiO_2_ anticaking agents exhibited a slight rise in hygroscopicity under both storage conditions, with TCP resulting in a more significant decrease than SiO_2_. It was evident from the results that the anticaking agents rendered their protective role through the formation of a moisture-protective barrier between powder particles, and this led to the prevention of moisture adsorption [7].

### 3.6. Rehydration Time and Rehydration Ratio

The results of rehydration time and rehydration ratio are given in Table 2. It is evident from the results that all parameters (storage temperature, anticaking agents, and storage duration) exhibited a significant (*p* < 0.05) influence on the powder rehydration time (PRT) and rehydration ratio (RR). There was an increasing trend in PRT in all samples, with a corresponding increase in the storage period. Initially under ambient storage conditions, the PRT of the control was found to be in the range of 1.42 ± 0.02 min to 2.23 ± 0.22 min, whereas samples under accelerated storage conditions showed the highest increase, ranging from 1.43 ± 0.02 min to 2.58 ± 0.03 min. This behavior may be ascribed to the reduced driving force responsible for moisture transfer with the progression of rehydration followed by the system markedly approaching equilibrium conditions [30]. Samples subjected to treatment by anticaking agents exhibited a slight increase in PRT, with a lower degree of increase observed for samples stored under ambient conditions (Table 2). Moisture gain was usually reduced by the anticaking agents during the storage period, leading to caking inhibition and greater surface-area provision for rehydration followed by PRT reduction [7].

All samples exhibited significant decreasing tendencies (*p* < 0.05) with respect to the rehydration ratio (RR) under storage duration; however, under accelerated storage conditions, the decreasing tendency was prominent in the case of control (3.65 ± 0.08) as compared to that of samples stored under ambient conditions (4.24 ± 0.02). Higher rehydration is indicative of a high degree of rehydration ability in water [31]; thus, it can be inferred that powder under ambient storage conditions exhibited an increased degree of rehydration ability. Under storage conditions, moisture was usually absorbed by the powder, and therefore, a reduction in driving forces responsible for water absorption occurred, which may have caused powder-rehydration difficulty.

### 3.7. Color Profile

The lightness (L*), redness or greenness (a*), and yellowness or blueness (b*) values of powder samples were significantly (*p* < 0.05) affected by storage conditions, storage period, and the anticaking agents. For control, under ambient conditions, the L* value was significantly (*p* < 0.05) reduced from 57.33 ± 3.13 to 50.27 ± 3.10, whereas under accelerated conditions, a more significant decrease was observed (57.33 ± 3.21 to 45.56 ± 3.11). TCP and SiO_2_ incorporation retained the lightness of the powder samples compared to control under both the storage conditions, with more retention under ambient conditions (Table 3). Amongst the anticaking agents, TCP was found to be more effective in retaining the lightness of the samples. The a* values of control under ambient and accelerated conditions increased with storage time from the initial value of 5.463 ± 1.06 to 8.429 ± 1.21 and 9.251 ± 1.06, respectively, thereby, increasing the redness of the samples (Table 3). However, the increase was more pronounced under accelerated conditions. A decrease in L* values and the increase in a* values was reported during storage owing to the browning reactions [32]. Similarly, Wong and Lim [33] reported similar findings for mango soy-fortified yogurt powder. Furthermore, samples treated with anticaking agents TCP and SiO_2_ under both the storage conditions showed a lesser increase in a* values compared to control, with a higher increase under accelerated storage conditions. Anticaking agents provide an encapsulating effect against moisture and oxygen, resulting in inhibition of browning reactions [7].

Furthermore, it can be observed from Table 3 that samples subjected to anticaking-agent treatment under both storage conditions had higher values of b* after completion of the storage period in comparison to control. However, it was highest (24.01 ± 2.23) in the sample treated with TCP compared to SiO_2_ under ambient conditions (Table 3). The retention of yellowness in the samples could be ascribed to the encapsulating effect of carriers against heat, light, and oxygen. Carotenoids impart a characteristic yellow color to apricot powder; however, carotenoids are susceptible to thermal treatment, oxygen, and light. These bioactive compounds can suffer auto-oxidation since their structural configuration has a conjugated double-bonding system spanning the whole polyene chain length [34].

### 3.8. Total Aerobic Bacteria (TAB) Count

The results of the TAB count are given in Table 3. It is evident from the results that all parameters, including storage temperature, anticaking agents, and storage duration, exhibited a significant (*p* < 0.05) influence on TAB count. Higher TAB counts were observed in the case of control samples subjected to both accelerated (7.98 × 10^3^ CFU/g) and ambient (5.60 × 10^3^ CFU/g) storage conditions. The safer limits of TAB, as prescribed by the Food Safety Standards Authority of India (FSSAI), the food regulatory authority of India, for food powders is 40,000 CFU/g [35]. Our results indicate that TAB in all the samples was quite lower than that prescribed by FSSAI and therefore were microbiologically safer. However, more TAB was found in the sample stored under accelerated conditions, with more observed in control (7.980 × 10^3^ CFU/g). This can be ascribed to the probable effect of the higher relative humidity (90 ± 2%) and storage temperature (40 ± 2 °C) during accelerated storage conditions, which may lead to the triggering of enhanced moisture transmission through ALP, thereby resulting in a_w_ rise in the samples and favoring microbial growth [36]. Since the water activity of the control under accelerated storage conditions was highest (0.881 ± 0.03) amongst all the samples, its highest TAB count is justified. The samples treated with anticaking agents, either TCP or SiO_2_, under both storage conditions had a TAB count within the permissible range (40,000 CFU/g) and hence, it can be inferred that these had high microbial safety and could be regarded as safe for consumption by the intended consumers.

### 3.9. Glass Transition Temperature (Tg) and Sticky-Point (Ts) Temperature

**Figure 2:**
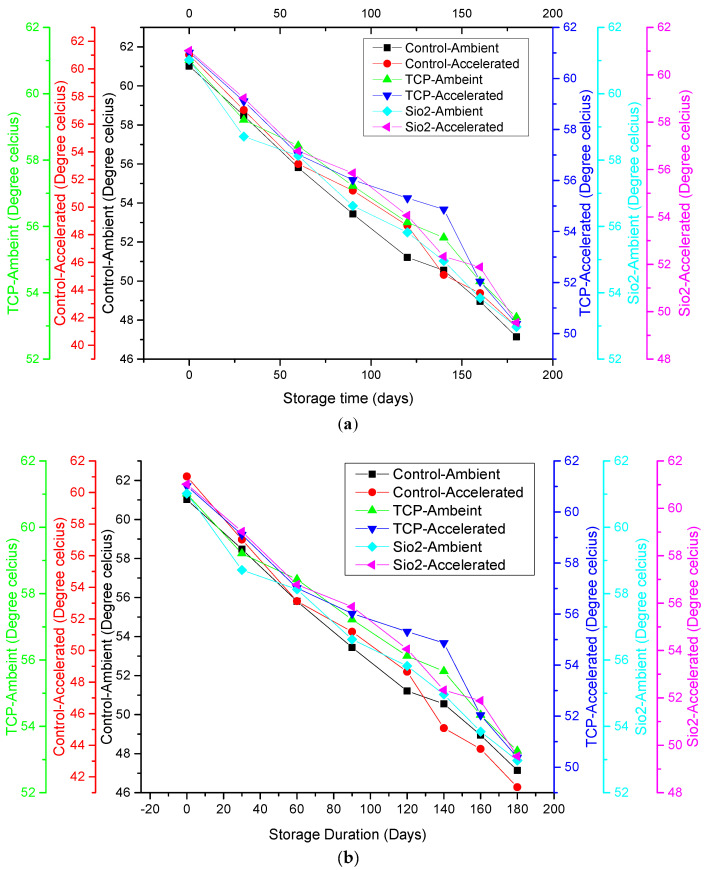
Relationship between (**a**) moisture gain and glass transition temperature of control and SDAP under ambient and accelerated storage conditions; (**b**) moisture gain and sticky-point temperature of control and SDAP under ambient and accelerated storage conditions.

As shown in Figure 2, at the commencement of storage, the powder samples, both treated and control, had a T_g_ of 57.80 ± 2.10 °C and a T_s_ of 61.02 ± 1.21 °C. This is indicative of the existence of a stable amorphous structural configuration in all samples, as the values indicate the presence of a glassy state in the powder samples owing to the T_g_ and T_s_ values being much higher than room temperature. The results depict that both T_g_ and T_s_ decreased with storage period in all the samples under both storage conditions, with a more significant decrease observed under ambient than accelerated storage conditions. The decreasing tendency in the case of T_g_ and T_s_ values might be attributed to the plasticizing effect of water, since water has a lower T_g_ value of −135 °C, which may have resulted in the decline in the amorphous material’s T_g_ during storage (Figure 2a,b) [8]. Furthermore, the consequential effect of moisture accumulation also caused enhanced molecule mobilization, exhibiting a greater tendency to undergo physical transition. Similarly, it was also reported by Fabra et al. [8] that the T_g_ and T_s_ for tomato and grapefruit powders also exhibited stepwise declining tendencies in a gradual manner as function of storage time.

Samples treated with the anticaking agents TCP and SiO_2_ showed a lesser decrease in T_g_ and T_s_. As indicated in Table 1, a lesser increase in moisture content was observed in TCP-treated samples than SiO_2_-treated samples, which is why a lesser decrease in T_g_ and T_s_ was observed in the former than the latter. Our results corroborated the findings of Barbosa-Cánovas et al. [7] well, who demonstrated the improved stability of powders by anticaking agents owing to rise in the product’s T_g_. Correspondingly, Phanindrakumar et al. [28] reported similar findings pertaining to pineapple powder. The temperature of the storage also influenced T_g_ and T_s_ as applied thermal treatment, resulting in the progression of physical transformation. Higher temperatures during accelerated storage conditions may have resulted in more physical transformation, and thus a more significant effect on T_g_ was observed. This agrees with the findings reported by Liu et al. [20] for tomato powder.

### 3.10. Storage-Life Prediction of Spray-Dried Apricot Powder Using GAB Model

**Figure 3:**
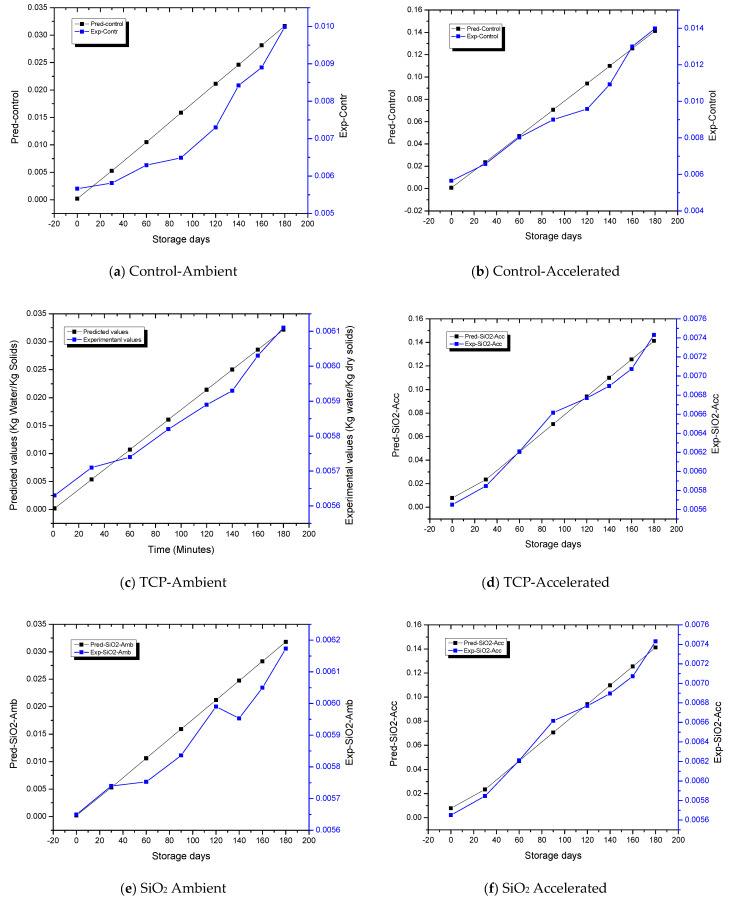
Graphical relationship between the time of storage and moisture content of spray-dried apricot powder.

Under both the ambient and accelerated storage conditions for control and treated ALP packaged samples, the initial value of water activity utilized was 0.28. Saturation vapor pressure of water at 25 °C (ambient) and 40 °C (accelerated) were acquired from steam-table data with p* as 3173.027 Pa at 25 °C and 7380.726 Pa at 40 °C, respectively. Storage-environment RH was taken as 0.60 (60%) for ambient and 0.90 (90%) for accelerated storage conditions, respectively. The pouch surface area was represented as A_p_ = 2 × 0.155 × 0.193 = 0.0600 m^2^, pouch water vapor permeability as K = 5.4 × 10^−8^ kg/m^2^/day/Pa, and dry solids amount present in 20 g of powder as W_s_ = 0.02 (1 − 0.004) = 0.0192 kg. Equation (4) elucidates the graphical relationship (Figure 3) between storage time and moisture content of apricot powder (kg water/kg dry apricot solids). Figure 3 also depicts the variation of experimental moisture content as a function of storage time. The predicted time required for moisture content of control samples subjected to ambient and accelerated conditions to rise from an initial value of X_i_ = 0.004 kg H_2_O/kg dry solids to the critical safe moisture content of powder value X_c_, taken as 0.006 kg H_2_O/kg dry solids, was 40 and 8 days, respectively, whereas the experimental time interval required for the moisture-content increase in powder was 45 and 16 days, respectively (Figure 3a,b). The predicted shelf life of TCP-treated samples under ambient and accelerated storage conditions was 157 and 77 days, respectively, whereas, the experimental time required for this increase in moisture content in powder was approximately 150 and 75 days, respectively (Figure 3c,d). Similarly, from Figure 3e,f, the predicted shelf life of SiO_2_-treated samples under ambient and accelerated storage conditions was 137 and 39 days, respectively, whereas the experimental time required for this moisture-content increase in the powder was 148 and 47 days, respectively. The predicted shelf life obtained from the GAB model and the experimental shelf life were very similar. The predicted and experimental moisture content exhibited a correlation coefficient of 0.981, indicating that the GAB model (Equation (4)) was adequate for predicting the shelf life of SDAP. The moisture gain had significant influence as a critical determinant of shelf life and quality of low-moisture food products. It has also been reported that quality deterioration usually increases dramatically when moisture content or a_w_ reached above certain levels and the time requirement for reaching to critical moisture content has been implied as an indicator of shelf life. The moisture-transfer rate and the extent in low-moisture foods packaged in plastic films may be subjected to variability depending on the a_w_ of food, storage conditions including humidity and temperature, and package permeability to water vapor.

## 4. Conclusions

Spray-dried apricot powder (SDAP) treated with either TCP or SiO_2_ when packed in an aluminum-laminated package and stored under ambient (25 ± 2 °C, 50 ± 2% RH) and accelerated (38 ± 2 °C, 90% RH) storage conditions for 180 days conclusively revealed that the SDAP could be better stored under ambient storage conditions for a period of six months without compromising much of its quality. Microbial count increased with the progression of storage; however, it was still well below the level of 40,000 CFU/g recommended by the FSSAI for fruit powders. Both TCP and SiO_2_ prevented the moisture ingress by providing the encapsulating effect, with the former being more effective than the latter. The results of this study also confirmed the feasibility of using the GAB model for predicting the shelf life of SDAP. The findings of this study will open up a new horizon in the field of packaging and post-harvest preservation of SDAP without compromising much of its quality in terms of both nutritional and microbial aspects.

## Figures and Tables

**Table 1:** Effect of storage duration, storage conditions (ambient and accelerated), and anticaking agents (TCP and SiO_2_) on moisture content, water activity, and degree of caking of spray-dried apricot powder (SDAP).

	Ambient			Accelerated		
Storage Duration (Days)	Control (0 kg/kg)	TCP (0.017 kg/kg)	SiO_2_ (0.017 kg/kg)	Control (0 kg/kg)	TCP (0.017 kg/kg)	SiO_2_ (0.017 kg/kg)
**Moisture Content (%)**						
0	5.613 ± 0.03 ^aAT^	5.613 ± 0.03 ^aAV^	5.613 ± 0.03 ^aAX^	5.613 ± 0.03 ^aAT^	5.613 ± 0.03 ^aAV^	5.613 ± 0.02 ^aAX^
30	5.813 ± 0.01 ^bAT^	5.710 ± 0.05 ^bBV^	5.740 ± 0.02 ^bCX^	6.567 ± 0.02 ^bAU^	5.862 ± 0.06 ^bBW^	5.846 ± 0.03 ^bCY^
60	6.290 ± 0.03 ^cAT^	5.743 ± 0.04 ^bBV^	5.753 ± 0.03 ^bBX^	8.028 ± 0.03 ^cAU^	5.940 ± 0.03 ^cBW^	6.210 ± 0.01 ^cCY^
90	6.487 ± 0.03 ^dAT^	5.826 ± 0.01 ^cBV^	5.836 ± 0.04 ^cBX^	8.997 ± 0.04 ^dAU^	6.136 ± 0.03 ^dBW^	6.616 ± 0.03 ^dCY^
120	7.300 ± 0.01 ^eAT^	5.891 ± 0.03 ^dBV^	5.990 ± 0.03 ^dCX^	9.580 ± 0.05 ^eAU^	6.276 ± 0.01 ^eBW^	6.770 ± 0.04 ^eCY^
140	8.421 ± 0.02 ^fAT^	5.936 ± 0.02 ^eBV^	5.953 ± 0.02 ^dBX^	10.930 ± 0.06 ^fAU^	6.632 ± 0.03 ^fBW^	6.896 ± 0.03 ^fCY^
160	8.902 ± 0.04 ^gAT^	6.033 ± 0.02 ^fBV^	6.050 ± 0.03 ^eBX^	11.002 ± 0.03 ^gAU^	6.853 ± 0.04 ^gBW^	7.073 ± 0.05 ^gCY^
180	9.987 ± 0.03 ^hAT^	6.113 ± 0.03 ^gBV^	6.173 ± 0.01 ^fCX^	11.990 ± 0.01 ^hAU^	7.273 ± 0.02 ^hBW^	7.430 ± 0.03 ^hCY^
**Water Activity (a_w_)**						
0	0.280 ± 0.03 ^aAT^	0.280 ± 0.02 ^aAV^	0.280 ± 0.02 ^aAX^	0.280 ± 0.03 ^aAT^	0.280 ± 0.04 ^aAV^	0.280 ± 0.08 ^aAX^
30	0.342 ± 0.02 ^bAT^	0.322 ± 0.07 ^bBV^	0.350 ± 0.04 ^bCX^	0.411 ± 0.07 ^bAU^	0.384 ± 0.03 ^bBW^	0.382 ± 0.07 ^bBY^
60	0.422 ± 0.05 ^cAT^	0.413 ± 0.03 ^cBV^	0.411 ± 0.03 ^cBX^	0.482 ± 0.03 ^cAU^	0.454 ± 0.03 ^cBW^	0.464 ± 0.03 ^cCY^
90	0.470 ± 0.03 ^dAT^	0.452 ± 0.01 ^dBV^	0.462 ± 0.05 ^dCX^	0.648 ± 0.05 ^dAU^	0.505 ± 0.02 ^BW^	0.515 ± 0.03 ^dCY^
120	0.542 ± 0.06 ^eAT^	0.505 ± 0.03 ^eBV^	0.522 ± 0.03 ^eCX^	0.692 ± 0.03 ^eAU^	0.553 ± 0.03 ^eBW^	0.566 ± 0.02 ^eCY^
140	0.570 ± 0.03 ^fAT^	0.533 ± 0.04 ^fBV^	0.593 ± 0.01 ^fCX^	0.742 ± 0.02 ^fAU^	0.621 ± 0.02 ^fBW^	0.613 ± 0.03 ^fCY^
160	0.601 ± 0.07 ^gAT^	0.574 ± 0.03 ^gBV^	0.621 ± 0.03 ^gCX^	0.807 ± 0.03 ^gAU^	0.642 ± 0.03 ^gBW^	0.654 ± 0.03 ^gCY^
180	0.710 ± 0.01 ^hAT^	0.601 ± 0.02 ^hBV^	0.634 ± 0.02 ^hCX^	0.881 ± 0.03 ^hAU^	0.720 ± 0.04 ^hBW^	0.731 ± 0.04 ^hCY^
**Degree of Caking (%)**						
0	10.191 ± 1.03 ^aAT^	10.191 ± 1.04 ^aBV^	10.191 ± 1.13 ^aCX^	10.191 ± 1.09 ^aAT^	10.191.03 ^aAV^	10.191 ± 1.05 ^aAX^
30	10.889 ± 1.03 ^bAT^	10.226 ± 1.03 ^bBV^	10.131 ± 1.23 ^bCX^	11.237 ± 1.03 ^bAU^	10.646 ± 1.06 ^bBW^	10.746 ± 1.03 ^bCY^
60	12.298 ± 1.01 ^cAT^	10.270 ± 1.03 ^cBV^	10.456 ± 1.07 ^cCX^	13.561 ± 1.05 ^cAU^	11.056 ± 1.06 ^cBW^	11.146 ± 1.07 ^cCY^
90	13.232 ± 1.04 ^dAT^	10.383 ± 1.04 ^dBV^	10.693 ± 1.07 ^dCX^	15.489 ± 1.03 ^dAU^	11.926 ± 1.03 ^dBW^	12.053 ± 1.03 ^dCY^
120	15.289 ± 1.07 ^eAT^	10.805 ± 1.07 ^eBV^	11.152 ± 1.06 ^eCX^	17.409 ± 1.03 ^eAU^	12.583 ± 1.01 ^eBW^	12.953 ± 1.04 ^eCY^
140	16.786 ± 1.03 ^fAT^	11.353 ± 1.08 ^fBV^	11.646 ± 1.09 ^fCX^	19.843 ± 1.01 ^fAU^	13.246 ± 1.03 ^fBW^	13.464 ± 1.04 ^fCY^
160	18.894 ± 1.07 ^gAT^	12.033 ± 1.03 ^gBV^	12.233 ± 1.04 ^gCX^	21.353 ± 1.01 ^gAU^	13.953 ± 1.02 ^gBW^	14.056 ± 1.02 ^gCY^
180	20.597 ± 1.03 ^hAT^	12.363 ± 1.07 ^hV^	13.036 ± 1.03 ^hCX^	24.761 ± 1.08 ^hAU^	14.803 ± 1.03 ^hBW^	15.242 ± 1.03 ^hCY^

Values are presented as mean ± standard deviation (S.D.). Values with different superscripts (small letters, a to h) within columns differ significantly (*p* < 0.05), representing the effect of storage days. Values with different superscripts (capital letters, A to C) within rows differ significantly (*p* < 0.05), representing the effect of anticaking agents within each storage condition. Values with different superscripts (capital letters, T to Y) within rows differ significantly (*p* < 0.05), representing the effect of storage conditions (ambient and accelerated).

**Table 2:** Effect of storage duration, storage conditions (ambient and accelerated), and anticaking agents (TCP and SiO_2_) on flowability, hygroscopicity, powder rehydration time, and rehydration ratio of spray-dried apricot powder (SDAP).

	Ambient			Accelerated		
Storage Duration (Days)	Control (0 kg/kg)	TCP (0.017 kg/kg)	SiO_2_ (0.017 kg/kg)	Control (0 kg/kg)	TCP (0.017 kg/kg)	SiO_2_ (0.017 kg/kg)
**Flowability (CI)**						
0	22.356 ± 2.03 ^aAT^	22.356 ± 2.05 ^aAV^	22.356 ± 2.01 ^aAX^	22.356 ± 2.13 ^aAT^	22.356 ± 2.11 ^aAV^	22.356 ± 2.08 ^aAX^
30	23.461 ± 2.07 ^bAT^	23.476 ± 2.03 ^bBV^	23.878 ± 2.03 ^bCX^	24.521 ± 2.23 ^bAU^	24.246 ± 2.04 ^bBW^	24.601 ± 2.01 ^bBY^
60	26.232 ± 2.03 ^cAT^	25.651 ± 2.03 ^cBV^	25.904 ± 2.02 ^cCX^	28.467 ± 2.03 ^cAU^	27.451 ± 2.03 ^cBW^	27.808 ± 2.03 ^cCY^
90	27.898 ± 2.05 ^dAT^	26.204 ± 2.02 ^dBV^	26.408 ± 2.03 ^dCX^	29.219 ± 2.33 ^dAU^	28.206 ± 2.06 ^dBW^	28.461 ± 2.02 ^dCY^
120	29.231 ± 2.03 ^eAT^	27.329 ± 2.03 ^eBV^	27.442 ± 2.01 ^eCX^	31.515 ± 2.04 ^eAU^	29.509 ± 2.03 ^eBW^	30.221 ± 2.03 ^eCY^
140	30.996 ± 2.01 ^fAT^	29.662 ± 2.07 ^fBV^	29.705 ± 2.03 ^fCX^	32.757 ± 2.09 ^fAU^	31.748 ± 2.08 ^fBW^	32.336 ± 2.05 ^fCY^
160	32.414 ± 2.03 ^gAT^	31.221 ± 2.03 ^gBV^	32.217 ± 2.05 ^gCX^	34.227 ± 2.03 ^gAU^	33.216 ± 2.24 ^gBW^	34.016 ± 2.03 ^gCY^
180	34.262 ± 2.02 ^hAT^	32.654 ± 2.09 ^hBV^	33.709 ± 2.08 ^hCX^	36.981 ± 2.31 ^hAU^	34.470 ± 2.25 ^hBW^	35.007 ± 2.23 ^hCY^
**Hygroscopicity (%)**						
0	23.041 ± 2.02 ^aAT^	23.041 ± 2.03 ^aAV^	23.041 ± 2.023 ^aAX^	23.041 ± 2.01 ^aAT^	23.041 ± 2.23 ^aBV^	23.041 ± 25 ^aCX^
30	23.253 ± 2.03 ^bAT^	23.173 ± 2.22 ^bBV^	23.192 ± 2.13 ^bCX^	23.767 ± 2.03 ^bAU^	23.471 ± 2.33 ^bBW^	23.527 ± 2.03 ^bCY^
60	23.577 ± 2.13 ^cAT^	23.321 ± 2.03 ^cBV^	23.371 ± 2.03 ^cCX^	24.656 ± 2.09 ^cAU^	24.021 ± 2.05 ^cBW^	24.132 ± 2.30 ^cCY^
90	24.053 ± 2.03 ^dAT^	23.867 ± 2.11 ^dBV^	24.027 ± 2.33 ^dCX^	25.062 ± 2.03 ^dAU^	24.773 ± 2.06 ^dBW^	25.073 ± 2.03 ^dCY^
120	25.223 ± 2.16 ^eAT^	24.073 ± 2.03 ^eBV^	24.671 ± 2.03 ^eCX^	26.137 ± 2.09 ^eAU^	25.247 ± 2.16 ^eBW^	26.023 ± 2.20 ^eCY^
140	26.232 ± 2.03 ^fAT^	24.784 ± 2.03 ^fBV^	25.107 ± 2.22 ^fCX^	28.232 ± 2.03 ^fAU^	26.477 ± 2.03 ^fBW^	27.067 ± 2.03 ^fCY^
160	27.109 ± 2.22 ^gAT^	25.439 ± 2.36 ^gBV^	25.957 ± 2.17 ^gCX^	29.553 ± 2.11 ^gAU^	27.666 ± 2.04 ^gBW^	28.223 ± 2.44 ^gCY^
180	28.432 ± 2.03 ^hAT^	26.023 ± 2.03 ^hBV^	26.223 ± 2.23 ^hCX^	31.247 ± 2.43 ^hAU^	28.289 ± 2.17 ^hBW^	29.027 ± 2.22 ^hCY^
**Powder Rehydration Time (PRT) (min.)**						
0	1.423 ± 0.13 ^aAT^	1.423 ± 0.02 ^aAV^	1.423 ± 0.02 ^aAX^	1.423 ± 0.05 ^aAT^	1.423 ± 0.04 ^aAV^	1.423 ± 0.21 ^aAX^
30	1.480 ± 0.22 ^bAT^	1.463 ± 0.14 ^bBV^	1.467 ± 0.03 ^bBX^	1.453 ± 0.23 ^bAU^	1.437 ± 0.06 ^bBW^	1.448 ± 0.27 ^bCY^
60	1.527 ± 0.44 ^cAT^	1.503 ± 0.13 ^cBV^	1.503 ± 0.11 ^cBX^	2.020 ± 0.14 ^cAU^	1.550 ± 0.11 ^cBW^	1.560 ± 0.21 ^cCY^
90	1.573 ± 0.56 ^dAT^	1.540 ± 0.02 ^dBV^	1.540 ± 0.23 ^dBX^	2.113 ± 0.03 ^dAU^	2.013 ± 0.03 ^dBW^	2.037 ± 0.27 ^dCY^
120	2.017 ± 0.12 ^eAT^	1.870 ± 0.05 ^eBV^	2.026 ± 0.07 ^eCX^	2.160 ± 0.16 ^eAU^	2.047 ± 0.15 ^eBW^	2.060 ± 0.36 ^eCY^
140	2.123 ± 0.03 ^fAT^	2.040 ± 0.04 ^fBV^	2.057 ± 0.22 ^fCX^	2.260 ± 0.18 ^fAU^	2.147 ± 0.03 ^fBW^	2.180 ± 0.55 ^fCY^
160	2.200 ± 0.17 ^gAT^	2.097 ± 0.13 ^gBV^	2.103 ± 0.44 ^gCX^	2.370 ± 0.14 ^gAU^	2.223 ± 0.19 ^gBW^	2.270 ± 0.44 ^gCY^
180	2.230 ± 0.22 ^hAT^	2.120 ± 0.23 ^hBV^	2.177 ± 0.21 ^hCX^	2.583 ± 0.13 ^hAU^	2.320 ± 0.18 ^hBW^	2.363 ± 0.28 ^hCY^
**Rehydration Ratio**						
0	6.047 ± 0.13 ^aAT^	6.047 ± 0.25 ^aAV^	6.047 ± 0.21 ^aAX^	6.047 ± 0.17 ^aAT^	6.047 ± 0.18 ^aAV^	6.047 ± 0.11 ^aAX^
30	5.447 ± 0.22 ^bAT^	5.481 ± 0.03 ^bBV^	5.474 ± 0.23 ^bCX^	5.417 ± 0.03 ^bAU^	5.463 ± 0.16 ^bBW^	5.453 ± 0.03 ^bCY^
60	5.413 ± 0.17 ^cAT^	5.483 ± 0.29 ^cBV^	5.457 ± 0.27 ^cCX^	5.127 ± 0.12 ^cAU^	5.351 ± 0.26 ^cBW^	5.421 ± 0.16 ^cCY^
90	5.371 ± 0.03 ^dAT^	5.453 ± 0.28 ^dBV^	5.376 ± 0.29 ^dCX^	5.033 ± 0.03 ^dAU^	5.181 ± 0.27 ^dBW^	5.295 ± 0.03 ^dCY^
120	5.310 ± 0.10 ^eAT^	5.393 ± 0.03 ^eBV^	5.288 ± 0.11 ^eCX^	4.971 ± 0.03 ^eAU^	4.991 ± 0.31 ^eBW^	5.021 ± 0.16 ^eCY^
140	5.211 ± 0.03 ^fAT^	4.557 ± 0.07 ^fBV^	5.235 ± 0.09 ^fCX^	4.541 ± 0.18 ^fAU^	4.463 ± 0.21 ^fBW^	4.722 ± 0.03 ^fCY^
160	4.553 ± 0.20 ^gAT^	4.752 ± 0.30 ^gBV^	4.812 ± 0.12 ^gCX^	4.053 ± 0.12 ^gAU^	4.223 ± 0.29 ^gBW^	4.242 ± 0.11 ^gCY^
180	4.247 ± 0.02 ^hAT^	4.843 ± 0.13 ^hBV^	4.671 ± 0.08 ^hCX^	3.653 ± 0.08 ^hAU^	3.863 ± 0.06 ^hBW^	3.763 ± 0.01 ^hCY^

Values are presented as mean ± standard deviation (S.D.). Values with different superscripts (small letters, a to h) within columns differ significantly (*p* < 0.05), representing the effect of storage days. Values with different superscripts (capital letters, A to C) within rows differ significantly (*p* < 0.05), representing the effect of anticaking agents within each storage conditions. Values with different superscripts (capital letters, T to Y) within rows differ significantly (*p* < 0.05), representing the effect of storage conditions (ambient and accelerated).

**Table 3:** Effect of storage duration, storage conditions (ambient and accelerated), and anticaking agents (TCP and SiO_2_) on color profile and total plate count (TPC) of spray-dried apricot powder (SDAP).

	Ambient			Accelerated		
Storage Duration (Days)	Control (0 kg/kg)	TCP (0.017 kg/kg)	SiO_2_ (0.017 kg/kg)	Control (0 kg/kg)	TCP (0.017 kg/kg)	SiO_2_ (0.017 kg/kg)
L*-Value						
0	57.337 ± 3.13 ^aAT^	57.337 ± 3.24 ^aAV^	57.337 ± 3.23 ^aAX^	57.337 ± 3.21 ^aAT^	57.337 ± 3.02 ^aAV^	57.337 ± 3.10 ^aAX^
30	57.304 ± 3.21 ^bAT^	57.327 ± 3.03 ^bBV^	57.323 ± 3.22 ^bCX^	56.323 ± 3.03 ^bAU^	56.383 ± 3.23 ^bBW^	56.330 ± 3.23 ^bCY^
60	56.902 ± 3.22 ^cAT^	57.267 ± 3.10 ^cBV^	57.215 ± 3.21 ^cCX^	54.102 ± 3.34 ^cAU^	55.615 ± 3.21 ^cBW^	55.530 ± 3.12 ^cCY^
90	55.346 ± 3.32 ^dAT^	56.967 ± 3.03 ^dBV^	56.933 ± 3.08 ^dCX^	52.217 ± 3.03 ^dAU^	54.867 ± 3.32 ^dBW^	54.946 ± 3.26 ^dCY^
120	53.779 ± 3.43 ^eAT^	55.428 ± 3.19 ^eBV^	55.348 ± 3.19 ^eCX^	50.789 ± 3.33 ^eAU^	53.803 ± 3.34 ^eBW^	53.680 ± 3.23 ^eCY^
140	52.121 ± 3.21 ^fAT^	54.873 ± 3.11 ^fBV^	54.677 ± 3.18 ^fCX^	48.668 ± 3.22 ^fAU^	53.293 ± 3.45 ^fBW^	53.246 ± 3.54 ^fCY^
160	51.223 ± 3.11 ^gAT^	54.247 ± 3.18 ^gBV^	54.071 ± 3.16 ^gCX^	46.243 ± 3.22 ^gAU^	52.647 ± 3.27 ^gBW^	52.543 ± 3.04 ^gCY^
180	50.278 ± 3.10 ^hAT^	53.993 ± 3.11 ^hBV^	53.285 ± 3.17 ^hCX^	45.561 ± 3.11 ^hAU^	51.653 ± 3.29 ^hBW^	50.821 ± 3.23 ^hCY^
a* Value						
0	5.463 ± 1.06 ^aAT^	5.463 ± 1.09 ^aAV^	5.463 ± 1.09 ^aAX^	5.463 ± 1.09 ^aAT^	5.463 ± 1.07 ^aAV^	5.463 ± 1.05 ^aAX^
30	5.964 ± 1.05 ^bAT^	5.896 ± 1.03 ^bBV^	5.946 ± 1.08 ^bCX^	6.019 ± 1.03 ^bAU^	5.641 ± 1.03 ^bBW^	5.743 ± 1.03 ^bCY^
60	6.412 ± 1.04 ^cAT^	6.357 ± 1.18 ^cBV^	6.280 ± 1.07 ^cCX^	6.673 ± 1.08 ^cAU^	6.853 ± 1.09 ^cBW^	6.757 ± 1.06 ^cCY^
90	7.017 ± 1.01 ^dAT^	7.007 ± 1.23 ^dBV^	6.940 ± 1.06 ^dCX^	7.227 ± 1.03 ^dAU^	7.287 ± 1.03 ^dBW^	7.296 ± 1.03 ^dCY^
120	7.323 ± 1.10 ^eAT^	7.223 ± 1.22 ^eBV^	7.047 ± 1.03 ^eCX^	7.661 ± 1.08 ^eAU^	7.627 ± 1.09 ^eBW^	7.583 ± 1.03 ^eCY^
140	7.816 ± 1.22 ^fAT^	7.547 ± 1.21 ^fBV^	7.357 ± 1.05 ^fCX^	8.232 ± 1.03 ^fAU^	8.037 ± 1.03 ^fBW^	8.057 ± 1.04 ^fCY^
160	8.012 ± 1.21 ^gAT^	7.943 ± 1.32 ^gBV^	7.862 ± 1.03 ^gCX^	8.767 ± 1.04 ^gAU^	8.343 ± 1.07 ^gBW^	8.372 ± 1.03 ^gCY^
180	8.429 ± 1.21 ^hAT^	8.023 ± 1.32 ^hBV^	8.123 ± 1.01 ^hCX^	9.251 ± 1.06 ^hAU^	8.853 ± 1.04 ^hBW^	8.712 ± 1.04 ^hCY^
b* Value						
0	27.128 ± 2.03 ^aAT^	27.128 ± 2.23 ^aAV^	27.128 ± 2.11 ^aAX^	27.128 ± 2.11 ^aAT^	27.128 ± 2.33 ^aAV^	27.128 ± 2.11 ^AX^
30	26.778 ± 2.17 ^bAT^	27.074 ± 2.43 ^bBV^	26.982 ± 2.03 ^bCX^	26.878 ± 2.16 ^bAU^	27.471 ± 2.03 ^bBW^	27.667 ± 2.03 ^bCY^
60	26.023 ± 2.03 ^cAT^	26.871 ± 2.21 ^cBV^	26.772 ± 2.11 ^cCX^	25.343 ± 2.03 ^cAU^	26.022 ± 2.21 ^cBW^	26.017 ± 2.22 ^cBY^
90	25.505 ± 2.03 ^dAT^	26.271 ± 2.43 ^dBV^	26.135 ± 2.03 ^dCX^	24.223 ± 2.23 ^dAU^	25.773 ± 2.03 ^dBW^	25.175 ± 2.34 ^dCY^
120	24.862 ± 2.22 ^eAT^	25.762 ± 2.12 ^eBV^	25.552 ± 2.03 ^eCX^	23.616 ± 2.03 ^eAU^	24.241 ± 2.21 ^eBW^	24.112 ± 2.03 ^eCY^
140	23.880 ± 2.03 ^fAT^	25.176 ± 2.25 ^fBV^	25.107 ± 2.21 ^fCX^	21.443 ± 2.23 ^fAU^	23.265 ± 2.03 ^fBW^	23.136 ± 2.03 ^fcY^
160	22.775 ± 2.027 ^gAT^	24.842 ± 2.21 ^gBV^	24.336 ± 2.23 ^gCX^	19.998 ± 2.03 ^gAU^	22.343 ± 2.31 ^gBW^	22.237 ± 2.21 ^gCY^
180	21.870 ± 2.03 ^hAT^	24.017 ± 2.23 ^hBV^	23.885 ± 2.23 ^hCX^	18.233 ± 2.21 ^hAU^	21.271 ± 2.03 ^hAW^	21.116 ± 2.29 ^hBY^
**Total Plate Count (TAB) (CFU/g × 10^3^)**						
0	0.661 ± 1.21 ^aAXT^	0.661 ± 1.21 ^aAV^	0.661 ± 1.27 ^aAX^	0.661 ± 1.21 ^aAT^	0.661 ± 1.28 ^aAV^	0.661 ± 1.53 ^aAX^
30	3.721 ± 1.22 ^bAT^	3.712 ± 1.55 ^bBV^	3.744 ± 1.53 ^bCX^	4.022 ± 1.24 ^bAU^	3.852 ± 1.53 ^bBW^	3.842 ± 1.10 ^bCY^
60	3.872 ± 1.24 ^cAT^	3.753 ± 1.21 ^cBV^	3.753 ± 1.53 ^cCX^	4.661 ± 1.53 ^cAU^	3.934 ± 1.26 ^cBW^	3.944 ± 1.53 ^cCY^
90	4.299 ± 1.53 ^dAT^	3.824 ± 1.53 ^dBV^	3.824 ± 1.11 ^dCX^	5.128 ± 1.27 ^dAU^	4.147 ± 1.53 ^dBW^	4.610 ± 1.53 ^dCY^
120	4.663 ± 1.27 ^eAT^	3.893 ± 1.21 ^eBV^	3.910 ± 1.53 ^eCX^	5.780 ± 1.53 ^eAU^	4.268 ± 1.27 ^eBW^	4.770 ± 1.10 ^eCY^
140	4.987 ± 1.53 ^fAT^	3.932 ± 1.53 ^fBV^	3.950 ± 1.26 ^fCX^	6.216 ± 1.28 ^fAU^	4.694 ± 1.53 ^fBW^	4.894 ± 1.19 ^fCY^
160	5.239 ± 1.29 ^gAT^	4.032 ± 1.23 ^gBV^	4.050 ± 1.29 ^gCX^	6.908 ± 1.28 ^gAU^	5.252 ± 1.29 ^gBW^	5.073 ± 1.53 ^gCY^
180	5.601 ± 1.53 ^hAT^	4.110 ± 1.19 ^hBV^	4.174 ± 1.18 ^hCX^	7.980 ± 1.57 ^hAU^	6.051 ± 1.33 ^hBW^	6.242 ± 1.10 ^hCY^

Values are presented as mean ± standard deviation (S.D.). Values with different superscripts (small letters, a to h) within columns differ significantly (*p* < 0.05), representing the effect of storage days. Values with different superscripts (capital letters, A to C) within rows differ significantly (*p* < 0.05), representing the effect of anticaking agents within each storage conditions. Values with different superscripts (capital letters, T to Y) within rows differ significantly (*p* < 0.05), representing the effect of storage conditions (ambient and accelerated).

## Data Availability

Data is available upon request from the corresponding authors.
